# Overexpression of Parkin Ameliorates Dopaminergic Neurodegeneration Induced by 1- Methyl-4-Phenyl-1,2,3,6-Tetrahydropyridine in Mice

**DOI:** 10.1371/journal.pone.0039953

**Published:** 2012-06-29

**Authors:** Minjuan Bian, Jie Liu, Xiaoqi Hong, Mei Yu, Yufang Huang, Zhejin Sheng, Jian Fei, Fang Huang

**Affiliations:** 1 State Key Laboratory of Medical Neurobiology, Shanghai Medical College, Fudan University, Shanghai, China; 2 School of Life Science and Technology, Tongji Universities, Shanghai, China; 3 Institutes of Brain Science, Fudan University, Shanghai, China; University G. D’Annunzio, Italy

## Abstract

Mutations in the *parkin* gene are currently thought to be the most common cause of recessive familial Parkinsonism. Parkin functions as an E3 ligase to regulate protein turnover, and its function in mitochondrial quality control has been reported recently. Overexpression of parkin has been found to prevent neuronal degeneration under various conditions both in vivo and in vitro. Here, we generated a transgenic mouse model in which expression of wild type parkin was driven by neuron-specific enolase (NSE) promoter. We reported that both young and old parkin transgenic mice exhibited less reduction of striatal TH protein and number of TH positive neurons in the substantia nigra induced by 1-Methyl-4-Phenyl-1,2,3,6-Tetrahydropyridine (MPTP), compared to wild type littermates. MPTP-induced mitochondrial impairment in the substantia nigra was improved in young parkin transgenic mice. Decreased striatal α-synuclein was demonstrated in old parkin transgenic mice. These results provide reliable evidence from the transgenic mouse model for parkin that overexpression of parkin may attenuate dopaminergic neurodegeneration induced by MPTP through protection of mitochondria and reduction of α-synuclein in the nigrostriatal pathway.

## Introduction

Parkinson’s disease (PD) is one of the most common neurodegenerative disorders characterized by selective and progressive loss of dopaminergic neurons in the substantia nigra pars compacta (SNpc) and the presence of cytoplasmic inclusions known as Lewy bodies (LB) in the surviving neurons [1]. Both environmental and genetic factors have been implicated in the development of sporadic or familial PD [2], [3], [4], however, the exact mechanisms underlying PD still remain obscure. Tremendous progress has been made over the past few years in discovering the genes linked to rare familial forms of PD. Mutations in α*-synuclein* [5], *parkin* [6], *DJ-1* [7], *PINK-1* [8], *UCH-L1* [9], *LRRK2* [10], *ATP13A2* [11], *Omi/HtrA2* [12] and *NR4A2* [13] have been reported to cause familial PD. Thus, the molecular pathways identified in the inherited forms of PD may provide vital clues on the pathogenesis of typical sporadic PD.

Mutations in the *parkin* gene are associated with autosomal recessive juvenile parkinsonism (AR-JP), a disease characterized by juvenile onset of Parkinsonian symptoms and pathology [6]. Parkin functions as an E3 ubiquitin ligase to mediate attachment of ubiquitin monomers or chains to substrate proteins; ubiquitination of substrates may undergo proteasomal degradation or have non-degradative functions [14], [15]. The abnormal accumulation and processing of mutant or damaged proteins which are normally targeted via ubiquitination to the proteasome have been implicated in many neurodegenerative diseases [16]. It has been hypothesized that familial PD associated mutations in parkin may result in loss of its E3-ligase activity and finally leads to the accumulation of non-ubiquitinated substrates, which is deleterious to the dopaminergic cells. Evidence from AR-JP brains have shown the accumulation of non-ubiquitinated forms of parkin substrates, such as Pael-R, cyclin E, CDCrel-1 and 2a, p38/AIMP2 and FBP1 [17], [18], [19], [20], [21], [22]. Previous studies have found that parkin can protect against toxicity induced by overexpression of relative substrates, such as mutant forms of α-synuclein [23], pael-R [18], [24] and mutant LRRK2 [25]. Parkin-Q311X mutant transgenic mice exhibit age-dependent dopaminergic neuron degeneration in substantia nigra (SN) and accumulation of α-synuclein in dopaminergic neurons [26]. All these work suggest a key role of parkin as an E3 ligase for dopaminergic neuron survival.

Evidence from PD brains provides supports to the notion of mitochondrial dysfunction and oxidative stress in the pathogenesis of PD [27], [28], [29]. Also, mitochondrial dysfunction and increased oxidative stress are obvious in parkin null mice and Drosophila [30], [31], [32]. Recent work reveals that parkin plays an important role in mitochondrial quality control by recognizing and eliminating damaged mitochondria from cells through mitophagy [33], [34]. PINK1, another causal gene for recessive familial forms of PD, may recruit parkin to the outer membrane of damaged mitochondria [35], [36], [37]. Parkin then regulates the removal of damaged mitochondria through proteasome- and mitophagy-dependent pathways [38], [39]. Acting in parallel to PINK1/ parkin pathway, DJ-1 also regulates mitochondrial function and mitophagy in the oxidative environment [40]. Overexpression of parkin can protect cells from mitochondrial dysfunction caused by either mitochondrial toxins [41], inactivation of PINK1 [42], [43], [44], [45] or DJ-1 [40]. Moreover, virus-mediated gene transfers of parkin in SN lead to neuroprotection against toxin 6-OHDA or MPTP [46], [47], [48], [49]. Taken together, these findings suggest that parkin functions as a protective agent through mitochondrial protection and overexpression of parkin may provide a novel therapeutic strategy for PD [50].

Therefore, in the present study, we tested whether overexpression of parkin executed dopaminergic neuroprotection against MPTP in parkin transgenic mice. We found that both young and old parkin transgenic mice exhibited less reduction of striatal TH protein and number of TH positive neurons in the SN induced by MPTP, especially in old transgenic mice. Furthermore, we revealed that MPTP-induced mitochondrial impairment in the SN was improved in parkin transgenic mice accompanied by elevated transcriptional expression of bcl-2 and DJ-1. Decreased striatal α-synuclein protein was shown in old parkin transgenic mice. These results provide the genetic evidence that overexpression of parkin may ameliorate MPTP-induced mitochondrial impairment and nigrostriatal α-synuclein levels, thus protect dopaminergic neurons from neurodegeneration.

## Methods

### Generation of Parkin Transgenic Mice

Mouse parkin cDNA was amplified from the brain by RT-PCR. For the 5′ terminal fragment of parkin, forward primer 5′ACCTGAATTCAGATCTCCCGGTGACCATGAT.

AGTGTTTGTC3′ and reverse primer 5′CTTCTCCAAGGATCCTGAAGTGATG3′ were used in PCR, EcoR I and BamH I restriction sites were underlined. For the 3′ terminal fragment of parkin, forward primer 5′CATCACTTCAGGATCCTTGGAGAAG3′ and reverse primer 5′GAACTCTAGAGAATTCTTACTTGTCATCGTCGTCCTTGTAC.

**Figure 1:**
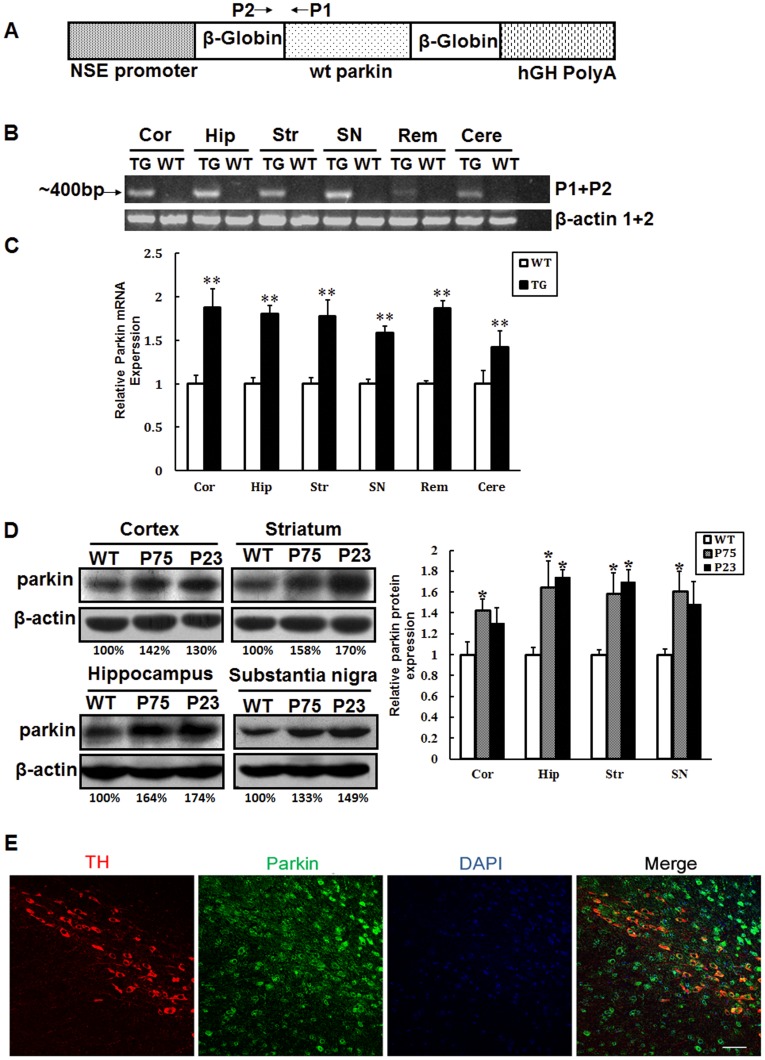
Generation and identification of parkin transgenic mice. (A) Schematic representation of the NSE-globin-parkin transgene construct. The expression of wild type parkin is under the control of NSE promoter. (B) Transcriptional expression of transgene in different regions of parkin transgenic mouse brain. The pair of primers P1 and P2 could identify mRNA expression (∼400 bp). Samples were from the cortex, hippocampus, striatum, substantia nigra, cerebellum and remaining of the brains from transgenic and wild type mice. (C) Transcriptional expression of parkin detected by real-time PCR in the cortex, hippocampus, striatum, substantia nigra, cerebellum and remaining parts of the brain, parkin-specific primers (parkin forward and parkin reverse) were used. (D) Expression of Parkin protein in the cortex, hippocampus, striatum and substantia nigra of wild type and two lines of transgenic mice. Transgenic mice showed increased parkin expression in these regions. Data presented are the means±SE. *p <0.05, **p<0.01, significant differences between wild type and transgenic mice; n = 3−7. (E) Colocalization of parkin and TH in the Substania nigra of parkin transgenic mice. Scale bar, 50 µm.

ACGTCAAACCAGTGATCTC 3′ were used in amplification, BamH I and Xba I restriction sites were underlined. The digested PCR products were cloned into pBluescript II SK between the EcoRI and Xba I sites and confirmed by sequencing. The full length parkin cDNA was cloned within β-globin sequence between the rat neuron-specific enolase (NSE) and human growth hormone polyA signal site. The final construct was digested with NotI and BssH II, a 6.8 kb transgene fragment was purified and microinjected into the pronucleus of fertilized eggs of C57BL/6×DBAF1 hybrid mice. Founders were backcrossed for six generations on a C57BL/6 background. The expression of the transgene was assessed by RT-PCR, in which the primers P1 (5′TGGCTGCTCAGGTCCACTCGTGTC 3′) and P2 (5′ CTTGGATCCTGAGAACTTC AGG 3′) (positions indicated in Figure 1A) could distinguish transgene mRNA expression (∼400 bp) from DNA amplification fragment (∼900 bp). Parkin mRNA and protein expression were assessed by quantitative Real-Time PCR and Western blot. Two lines of parkin transgenic mice were used in this study (Line P75# and Line P23#). After confirming the protective effects on dopaminergic neurons against MPTP within these two lines of transgenic mice, most of the experiments were conducted on mice of Line P75# if not mentioned. All the mice were maintained in the specific pathogen-free facility at Fudan University. All mouse care and experimentation were approved by the Institutional Animal Care and Use Committee of Fudan University Shanghai Medical.

College (IACUC Animal Project Number: 20080307-055). All surgery was performed under chloral hydrate anesthesia, and all efforts were made to minimize suffering.

### MPTP Treatment and Tissue Preparation

Young (9–12 weeks old) and old (11–13 months old) male transgenic and littermate wild type mice were injected intraperitoneally with MPTP or 0.9% saline for 2 days with 2 injections at 12 h interval within a day. The dosage of MPTP was 20 mg/kg for young mice and 15 mg/kg for old mice. The mice were sacrificed at 1 day or 3 days after the last MPTP administration. Mice were anesthetized with 10% chloral hydrate to minimize suffering, and then perfused intracardially with 0.9% saline solution. Brains were carefully removed and subdivided for different experimental procedures. For some mice, the right cerebral hemispheres were used for section preparation, and the striatum and SN isolated from the left cerebral hemispheres were applied for protein preparation and total RNA extraction, respectively. For other mice, SNpc were microdissected for electron microscopy assay.

### Western Blot Analysis

Dissected mouse brain tissues were lysed in RIPA buffer [50 mM Tris-HCl (pH 7.5), 150 mM NaCl, 1% NP-40, 0.5% sodium deoxycholate, and 0.1% sodium dodecyl sulfate] containing complete protease inhibitor cocktail (Calbiochem, San Diego, USA). 25 µg protein of each sample was loaded on SDS-PAGE gels and transferred to polyvinylidene difluoride membranes (Schleicher and Schuell, Dassel, Germany). The membranes were blocked by 5% non-fat dried milk in TBS-T [10 mM Tris-HCl (pH 8.0), 150 mM NaCl, and 0.1% Tween], and sequentially incubated with the following primary antibodies: mouse anti-tyrosine hydroxylase (1∶4000; Sigma, USA); mouse anti-β-actin (C4) (1∶500; Santa Cruz, USA); mouse monoclonal Hsp70 antibody (1∶400; Santa Cruz, USA); rabbit anti-α-synuclein (1∶2000; Sigma, USA); Parkin, Bax, Bcl-2 and UCH-L1 (1∶1000; Cell signaling technology, USA); PINK1 and DJ-1(1∶1000; Abcam, USA), and then with peroxidase-conjugated anti-rabbit or anti-mouse immunoglobulin G (1∶10,000 dilution in TBS-T). Signals were detected with a chemiluminescence detection system (Santa Cruz, USA). The protein levels were quantified by densitometry analysis using Quantity One 4.5.2 software (Bio-Rad, Hercules, USA).

### Immunohistochemistry and Immunofluorescence

The right cerebral hemispheres were post-fixed in 4% paraformaldehyde in 0.1 M phosphate buffer (pH 7.2), and then stored in a 30% sucrose solution for 24–48 h at 4°C until they sank. Frozen sections were then cut at 30 µm on freezing microtome (Leica, Germany). Immunohistochemistry of brain tissues was carried out according to previously published methods [51] with minor modifications. Briefly, sections were placed in blocking buffer containing 10% goat serum with 0.3%Triton X-100 in 0.01 M phosphate buffered saline (pH 7.2) at 37°C for 35 min. They were then incubated at 37°C for 2 hr with mouse anti-tyrosine hydroxylase (1∶2000) in PBS containing 1% goat serum and 0.3% Triton X-100. Sections were then incubated with biotinylated anti-mouse secondary antibody (1∶200; Vector Laboratories, USA) at 37°C for 30 min and avidin-biotin-peroxidase (1∶200) at 37°C for 45 min. The peroxidase reaction was detected with 0.05% DAB (Sigma, USA) in 0.1 M Tris buffer and 0.03% H_2_O_2_. For immunofluorescence, sections were blocked and then incubated at 37°C for 2 hr with mouse anti-tyrosine hydroxylase (1∶2000) and rabbi anti-parkin (1∶200; Abgent, USA). Sections were then incubated with Alexa Fluor 488 conjugated goat anti-rabbit and with Alexa Fluor 594 conjugated donkey anti-mouse secondary antibodies (1∶500; Invitrogen, USA) at 37°C for 45 min. Then the sections were mounted with ProLong® Gold Antifade Reagent with DAPI (Invitrogen, USA) and images were obtained under a Leica confocal microscope (TCS SP-2, Leica, Germany).

### Densitometric Analysis

The densitometric analysis of TH positive fiber of the striatum was done as previously described [52]. Briefly, An average of 4 section of the striatum starting from the rostral anteroposterior (+1.60 mm) to anteroposterior (0.00 mm), according to bregma of the brain atlas, were examined at a ×5 magnification using the IMAGE PRO PLUS system(vision 6.0; Media Cybernetics) on a computer attached to a light microscope (Leica, Germany). To determine the density of the TH-immunoreactive staining in the STR, a square frame of 700×700 mm was placed in the dorsal part of the STR. A second square frame of 200×200 mm was placed in the region of the corpus callosum to measure background values. To control for variations in background illumination, the average of the background density readings from the corpus callosum was subtracted from the average of density readings of the STR for each section. Then, the average of all sections of each animal was calculated separately before the data were processed statistically.

### Cell Counting

For measurement of the density of TH positive cells in the substantia nigra pars compacta, we performed stereological counting based on the previous description [53]. Total numbers of TH-positive neurons and Nissl-stained neurons in SNpc were counted stereologically using the optical fractionator method on a Stereo Investigator (Micro Brightfield, USA) system, which is attached to a Leica microscope. Briefly, one out of five 30 µm-thick sections and totally four sections from bregma −2.80 to −3.65 mm were collected. The SN was delineated at a ×5 objective, and the actual counting was performed under a ×20 objective. The stereological counting was performed in a double-blind fashion (n = 3−5 per group).

### Electron Microscopy

3 days after the last MPTP or saline administration, substantia nigra pars compacta from young transgenic and wild type mice was isolated and fixed in 2.5% glutaraldehyde in phosphate buffered solution at 4°C for 18 hr, and then post-fixed in 1% osmium tetroxide. After extensive washing in distilled water, the samples were dehydrated in a graded series of alcohols, embedded in an Epon-Araldite mixture, and polymerized at 60°C for 48 hr. 70 nm ultrathin sections were observed with a Philips CM120 electron microscope. Mitochondrial number and morphology were determined in images from 3–5 different fields in each group (n = 3). 90–135 mitochondria were measured in neurons per group.

### Quantitative Real-Time PCR

Total RNAs were extracted from the substantia nigra using TRIzol reagent (Invitrogen, USA). Reverse transcription was carried out using random primers and Moloney murine leukemia virus reverse transcriptase (Promega, USA). Real-time PCR was conducted for quantification of parkin, bcl-2, bax, PINK1, DJ-1, Timm22 and GAPDH mRNA on ABI 7300 PCR machine (Applied Biosystems, USA). For plotting a standard curve, serially diluted cDNA fragments were used in each experiment. Expression of target gene or GAPDH was quantified to the standard curve, and the relative expression value was calculated as the ratio of target cDNA to GAPDH. Each of these reactions was carried out in duplicates. The primers used in the real-time PCR were:

Parkin forward: 5′CCAAACCGGATGAGTGGTGAGTGC3′;

Parkin reverse: 5′ACACGGCAGGGAGTAGCCAAGTTG3′;

bcl-2 forward: 5′GGATTGTGGCCTTCTTTGAGTTCGG3′;

bcl-2 reverse: 5′CATATTTGTTTGGGGCAGGTTTGTC3′;

bax forward: 5′GCGTGGTTGCCCTCTTCTACTTTGC3′;

bax reverse: 5′GAAGAAAAGACACAGTCCAAGGCAG3′;

DJ-1 forward: 5′GCTTCCAAAAGAGCTCTGGTCATCC3′;

DJ-1 reverse: 5′CAGATGGCAGCTATGAGGCCCTTCC3′;

PINK1 forward: 5′CTTATAGGAAAGGGCCCGGATGTCG3′;

PINK1 reverse: 5′GATGATGTTAGGGTGTGGGGCAAGC3′;

Timm22 forward: 5′CGAGGAGCAGAAGATGATCGAGAGG3′;

Timm22 reverse: 5′GATGACGCTGTTCTTCCAGTCCGAC3′;

GAPDH forward: 5′GTAGACAAAATGGTGAAGGTCGGTG3′;

GAPDH reverse: 5′CTCGCTCCTGGAAGATGGTGATGGG3′.

### Data Analysis

Data were analyzed using SPSS software (version 11.5, USA). All values were expressed as means ± SE. Statistical analysis of group differences was assessed by ANOVA followed by multiple comparisons with the LSD post-hoc test. *P*<0.05 was considered significant.

## Results

### Generation and Identification of Parkin Transgenic Mice

The DNA construct used for generation of parkin transgenic mice was schematically depicted in Figure 1A. For selective expression of parkin in neurons, mouse wild type parkin cDNA was put under the rat neuron-specific NSE promoter [54], [55], [56]. By RT-PCR, we could detect the expression of parkin transgene in the brains of transgenic mice (data not shown). Moreover, the parkin transgene was expressed widely in different brain regions, including the cortex, hippocampus, striatum, substantia nigra, cerebellum and the remaining part (Figure 1B). The Real-time PCR results showed about 0.5∼0.8 fold elevated parkin mRNA expression in these brain regions in the transgenic mice (Figure 1C). By immunoblotting, 0.3∼0.6 fold elevated parkin protein in the cortex, hippocampus, striatum and substantia nigra was observed in the transgenic mice (Figure 1D). Confocal images showed parkin colocalized with TH in the substantia nigra pars compacta in transgenic mice (Figure 1E). The transgene did not have any obvious effect on breeding and the gross brain morphology (data not shown).

### Parkin Attenuates MPTP Induced Dopaminergic Neurondegeneration

**Figure 2:**
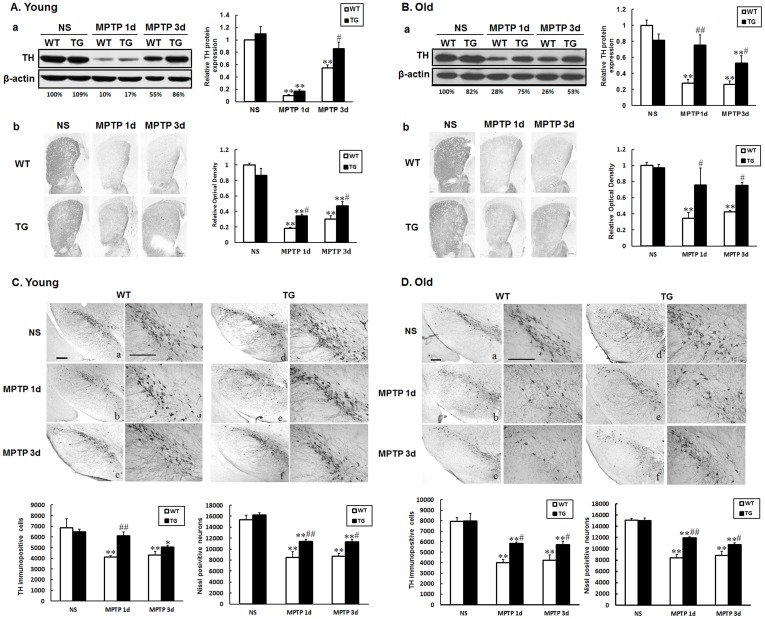
MPTP elicited less dopaminergic toxicity in parkin transgenic mice. Samples were collected at 1 day or 3 days from the striatum (A, B) and the substantia nigra (C, D) after saline or MPTP treatment. Western blot showing striatal TH protein expression in young (A-a) and old (B-a) mice. Quantification of relative TH protein expression was showed in the right panel. Immunohistochemical staining of the striatum showing TH positive nerve fibers of young (A-b) and old (B-b) mice. Scale bar, 0.2 mm. Relative optical density of the staining was showed in the right panel. Immunohistochemical staining showing TH positive cells in the substantia nigra of young (C) and old (D) mice. The sections were from wild type (a, b, c) or parkin transgenic (d, e, f) mice; (a, d) saline; (b, e) 1 day after MPTP treatment; (c, f) 3 days after MPTP treatment. Images to the right are higher magnification of the SN sections. Statistical data of the number of TH positive neurons (left panel) and nissl positive neurons (right panel) were showed in the bottom. Scale bar, 20 µm. Data presented are the means±SE. *p <0.05 and * *p <0.01, significant differences between saline and MPTP-treated mice; #p<0.05 and # #p<0.01, significant differences between wild type and transgenic mice; n = 3−6.

1 day or 3 days after the last MPTP injection, we observed significant depletion of striatal TH protein both in young and old wild type mice (Figure 2A, B). However, both young and old parkin transgenic mice revealed striatal TH protein reduction to a less extent. The rescue of striatal TH protein was especially obvious in young transgenic mice 3 days after the last MPTP administration, and in old ones at both experimental time points (Figure 2 A-a, B-a). By western blot, in young mice, we found that MPTP elicited about 90% (1 day afterwards) to 55% (3 days afterwards) decrease of striatal TH protein in wild type mice and 82% (1 day) to 15% (3 days) reduction of TH protein in transgenic mice. While in old mice, MPTP elicited nearly 70% (1 day or 3 days afterwards) decrease in wild type mice and 25% (1 day) to 50% (3 days) reduction in transgenic mice (Figure 2A-a, B-a). On the 8th day, MPTP elicited 57% decrease of striatal TH protein in wild type mice and only 30% reduction of TH protein in transgenic mice (Figure S1). Immunostaining of TH positive nerve fibers in the striatum showed similar results to the western blot analysis of striatum protein. The losses of TH positive fibers were about 80% and 70% in wild type young mice 1day or 3days after MPTP intoxication. The values were about 65% (1day) to 50% (3days) in transgenic mice. While in old ones, MPTP elicited about 66% (1day) to 58% (3days) TH positive fibers in wild type mice and about 25% (1day or 3days) in transgenic mice (Figure 2A-b, B-b). By immnunostaining, we found that in the SN, TH positive neurons showed a similar tendency to the TH protein in the striatum. MPTP elicited about 41% (1 day) to 38% (3 days) (Figure 2C-b, c) and 50% (1 day) to 53% (3 days) (Figure 2D-b, c) loss of TH neurons in the SNpc in young and old wild type mice, respectively. Prominent decrease of TH neuron loss induced by MPTP was observed in young transgenic mice 1 day afterwards (Figure 2C-e) or in old transgenic mice both 1day and 3 days after the last MPTP administration (Figure 2D-e, f). By counting Nissl stained neurons in the SN of wild type and transgenic mice, we observed the loss of Nissl positive neurons after MPTP intoxication, but the transgenic mice showed less reduction of neurons in both young and old groups (Figure 2C, D). This phenomenon parallels to the TH neuron counting, which confirms that parkin transgene rescues the dopaminergic neuronal loss rather than a loss of TH phenotype. All together, MPTP elicited less dopaminergic toxicity in parkin transgenic mice. The attenuated MPTP dopaminergic toxicity was confirmed in a second line of parkin transgenic mice (P23#). Both young and old transgenic mice showed less striatal TH protein depletion 1 day after MPTP administration compare to their wild type littermates (Figure S2).

### Parkin Improves Morphological Impairment of Mitochondria Induced by MPTP

**Figure 3:**
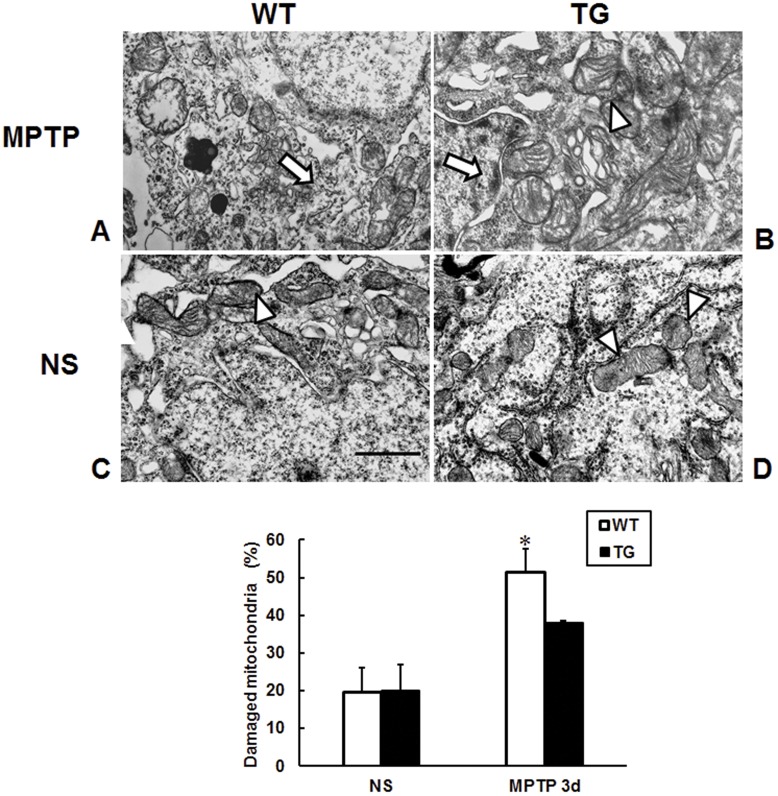
Less morphological damage of mitochondria in the SNpc neurons of parkin transgenic mice induced by MPTP. Electron microscopy showing the morphology of mitochondria in the SNpc from young mice at 3 days after MPTP (A, B) or saline treatment (C, D). (A, C) wild type mice; (B, D) parkin transgenic mice. Arrows represent abnormal mitochondria with numerous vacuoles and fragmented cristae; Triangles represent normal mitochondria. (E) Quantitative analyses of the percentage of damaged mitochondria in the SNpc neurons. Scale bar, 1 μm; Data presented are the means±SE. *p <0.05, significant differences between saline and MPTP-treated mice; n = 3 per group.

Several lines of evidence have indicated that overexpression of parkin may diminish mitochondrial dysfunction induced by various insults [41], [42]. In order to examine whether parkin could protect mitochondria from MPTP intoxication, we observed morphology of mitochondria in the SNpc 3 days after MPTP administration in young mice. Electron microscopic analyses revealed that MPTP induced a significant higher number of structurally altered mitochondria in SNpc neurons of wild type mice, these alterations comprised numerous vacuoles and fragmented cristae (Figure 3A), while less deformed mitochondria were observed in parkin transgenic mice (Figure 3B). By quantification, wild type mice after MPTP administration exhibited significantly higher percentage of damaged mitochondria (51.3%) than saline-treated mice (Figure 3C, D), while lower percentage (37.8%) of damaged mitochondria was found in the SNpc of parkin transgenic mice after MPTP treatment (Figure 3E).

### Parkin Increases the Transcriptional Expression of bcl-2 and DJ-1 in the SN

**Figure 4:**
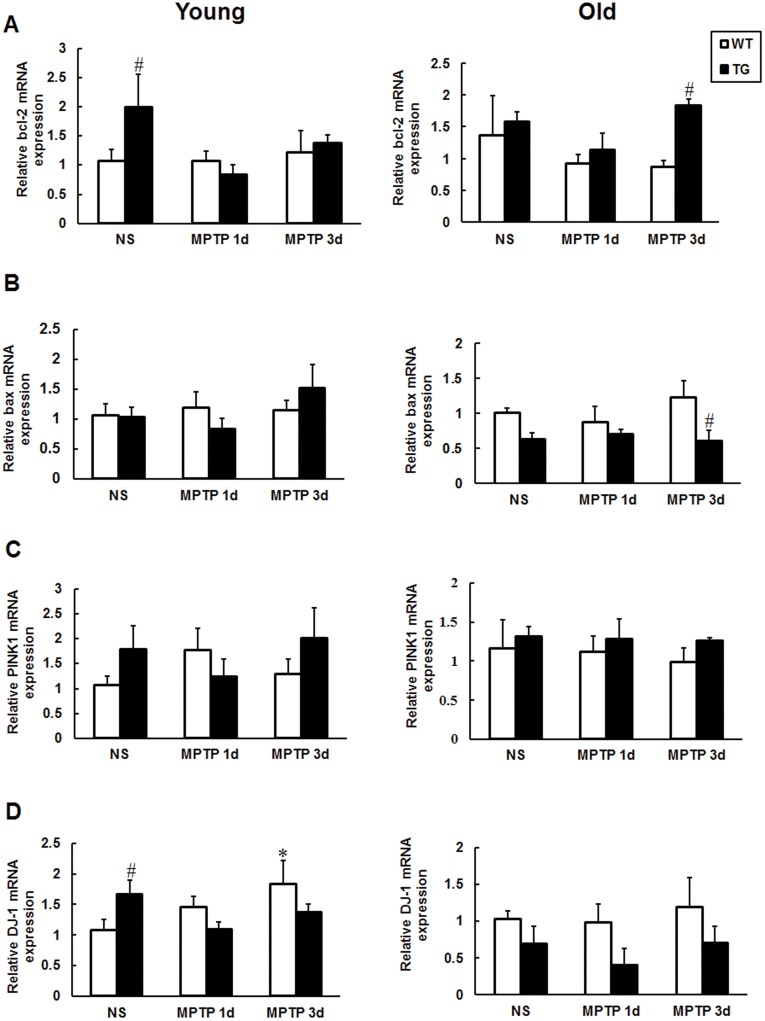
Transcriptional expression of bcl-2, bax, PINK1 and DJ-1 in the substantia nigra. Samples were from young and old mice treated with MPTP or saline. mRNA expression levels of bcl-2 (A), bax (B), PINK1 (C), DJ-1 (D) were determined by real-time PCR and normalized to GAPDH. Values are means±SE. *p <0.05, significant differences between saline and MPTP-treated mice; #p<0.05, significant differences between wild type and transgenic mice; n = 4−7 per group.

Transcriptional expression of anti-apoptotic molecule bcl-2, pro-apoptotic molecule bax and mitochondria related gene PINK1, DJ-1 in the SN were detected by real-time PCR. We found that bcl-2 and DJ-1 up-regulated in the young parkin transgenic mice compared to the wild type littermates, while MPTP administration deprived the elevation of bcl-2 and DJ-1 expression at the two selected time points (Figure 4A, D). The up-regulation of bcl-2 and down-regulation of bax were detected in the old parkin transgenic mice 3 days after MPTP injection (Figure 4A, B). These data may in part explain the beneficial roles of parkin in young and old transgenic mice. No significant alteration of bax and PINK1 mRNA was observed in young mice within groups (Figure 4B, C); neither were PINK1 and DJ-1 mRNA in old mice (Figure 4C, D). Timm22, a non-affected gene in MPTP induced PD model, showed no changes (Figure S3E). At 1day after MPTP administration, the transcriptional expression of iNOS was increased in young wild type mice, but reduced significantly in littermate transgenic mice (Figure S3A). At 3 days afterwards, the transcription of CHIP was dramatically decreased in old transgenic mice (Figure S3C). TNF-α and UCH-L1 had no significant changes among experimental groups (Figure S3B, D).

### Parkin Reduces Striatal α-synuclein Protein Expression, but not Hsp70 Expression

**Figure 5:**
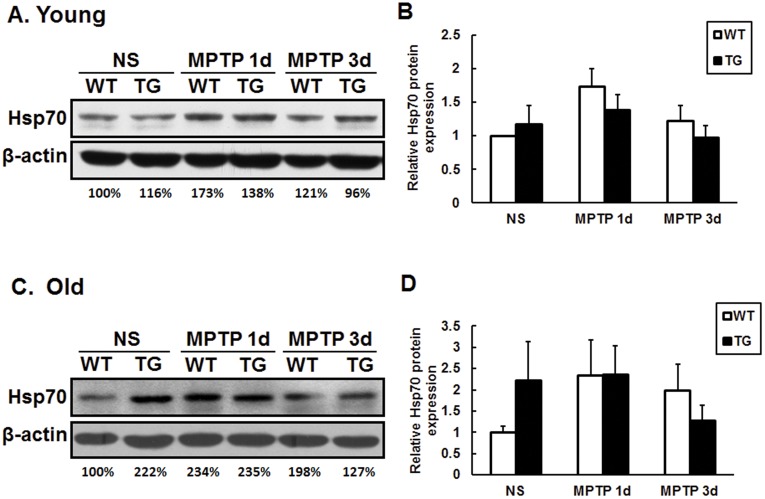
No changes of Hsp70 protein expression in the striatum of wild type and parkin transgenic mice. Western blot showing Hsp70 protein in the striatum of young (A) and old (C) mice after saline or MPTP treatment. Quantification of relative Hsp70 protein expression shown in B, D. Data presented are the means±SE. n = 4−6 per group.

**Figure 6:**
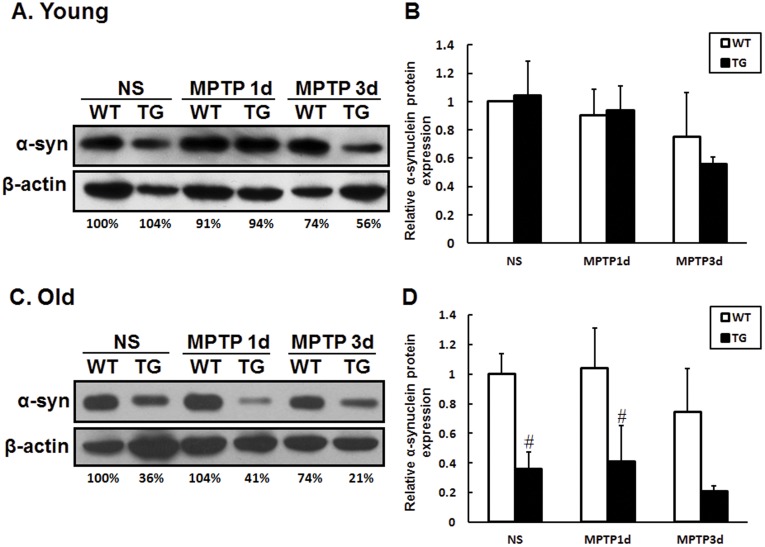
Levels of α-synuclein protein expression in the striatum of wild type and parkin transgenic mice. Western blot showing α-synuclein protein in the striatum of young (A) and old (C) mice after saline or MPTP treatment. (B, D) Quantification of relative α-synuclein protein expression. Data presented are the means±SE. # p<0.05, significant differences between wild type and transgenic mice; n = 4−6 per group.

As one of the substrate proteins of parkin, Hsp70 is reported to exert anti-apoptotic activity by blocking the function of several key proapoptotic factors [57], [58]. Thus, we observed the expression of Hsp70 protein in the striatum within groups and couldn’t find any significant changes both in young and old mice (Figure 5). α-synuclein is involved in the formation of Lewy Body in PD [59]. In our experiments, saline or MPTP-treated young mice exhibited similar level of α-synuclein protein in the striatum of both transgenic mice and wild type littermates (Figure 6A, B). Interestingly, striatal α-synuclein protein was dramatically decreased in old parkin transgenic mice with saline or MPTP treatment (Figure 6C, D). The striatal Bax, Bcl-2, PINK1, DJ-1, UCH-L1 protein had no significant alteration among all experimental groups (Figure S4).

All the results in the striatum and SN of wild type and Parkin transgenic mice were summarized in Table S1.

## Discussion

The major finding of this study is that overexpression of parkin reduces MPTP-induced dopaminergic toxicity. We have generated parkin transgenic mice in which the expression of parkin is controlled by neuron-specific enolase promoter and found that compared to wild type littermates; parkin transgenic mice are relatively resistant to MPTP, especially the old transgenic mice. Further evidences reveal that parkin may ameliorate impairment of mitochondria induced by MPTP and reduce striatal α-synuclein protein, and finally protect dopaminergic neurons against MPTP. The in vivo genetic model we used is more convenient and consistent for investigation of mechanisms underlying PD process under the condition of parkin overexpression, and our experiments were systematically conducted both in young and old mice.

Previous studies have described the neuroprotection of parkin against various insults in vitro or in vivo, such as Pael-R or mutant α-synuclein overexpression [18], [23], [24], excitotoxicity [20] and mitochondrial toxins [41]. Gene transfer of parkin in the SN mediated by viral vectors are reported to show protection of dopaminergic system against MPTP or 6-OHDA [46], [47], [48]. Our data, in accordance with previous findings, demonstrate less dopaminergic toxicity induced by MPTP in parkin transgenic mice. After MPTP administration, striatal TH expression showed robust reduction at 1 day and 3 days in young wild type mice, while overexpression of parkin didn’t rescue TH reduction at 1 day, but showed protective effect at 3 days (Figure 2A, B). In the old mice, the protective effect of parkin overexpression was found at both time points (Figure 2A, B). The results of striatum TH positive nerve fiber immunostaining parallel to the western blot results. Significant decreases in the loss of TH positive cells were observed in the SNpc of young transgenic mice at 1 day (Figure 2C) or old transgenic mice both 1day and 3 days after the last MPTP administration (Figure 2D). The different reaction patterns towards MPTP administration in the striatum and substantia nigra of young or old mice may be due to different doses of MPTP or different sensitivity to the toxin between dopaminergic cell bodies in the SN and terminals in the striatum [60].

Mitochondrial dysfunction has been implicated in the SNpc of PD patients [61], [62], [63] as well as parkin deficient Drosophila and mice [30], [31]. Thus, we hypothesize that parkin might prevent dopaminergic neurons from neurodegeneration through mitochondrial protection. The result showed that MPTP-induced abnormal mitochondria with numerous vacuoles and fragmented cristae were diminished in parkin transgenic mice (Figure 3B), demonstrating the mitochondrial protection of parkin against MPTP. This is further supported by elevated transcriptional expression of bcl-2 and DJ-1 in the SN of young parkin transgenic mice (Figure 4A, D). PINK1, the upstream molecule of parkin [44], did not exhibit mRNA level changes within groups. As a prime inhibitor of mitochondrial-dependent apoptosis [64], Bcl-2 overexpression protects dopaminergic neurons against neurotoxins [65], [66]. Overexpression of DJ-1 also protects cells against mitochondrial complex 1 inhibitors and oxidative stress induced by hydrogen peroxide [67], [68]. iNOS is important in the MPTP neurotoxic process [69], and the upregulation of iNOS mRNA in MPTP-injected wild type mice at 1 d afterwards was inhibited in Parkin transgenic mice. However, whether parkin directly or indirectly regulates the transcriptional expression of bcl-2, DJ-1 and iNOS remains to be explored.

A robust age-related increase in α-synuclein protein within nigral dopaminergic neurons during normal aging have been revealed previously [70], [71]. Lines of evidence has indicated that increase in wild type or mutant α-synuclein can be injurious to neurons [72], [73], [74] by leading to formation of intraneuronal aggregates and reduction of dopaminergic terminals [75] or causing more vulnerability of SNpc neurons to MPTP [76].

Hsp70 is one of the molecular chaperones that facilitate refolding misfolded proteins or help abnormal proteins for proteasomal degradation [77]. Parkin or Hsp70 expression can prevent α-synuclein aggregation and toxicity both in vitro and in vivo [23], [78], [79], [80]. Accumulation of proteinase K-resistant endogenous α-synuclein in SN has shown in parkin-Q311X mutant transgenic mice [26]. In current study, Hsp70 protein displayed no significant changes within groups (Figure 5). Interestingly, remarkable reduction of striatal α-synuclein was observed in old parkin transgenic mice (Figure 6). Our results manifest that overexpression of parkin and diminished α-synuclein-induced neurotoxicity might facilitate dopaminergic neurons against MPTP, especially in old transgenic mice. The difference of striatal α-synuclein levels and transcriptional expression of bcl-2, bax, DJ-1 (Figure 4), iNOS (Figure S3A) in young and/or old transgenic mice clearly indicates multiple mechanisms underlying the neuroprotection of parkin in young and old mice.

Taken together, this study identifies less MPTP neurotoxicity in parkin transgenic mice, suggesting the protective functions of parkin in nigrostriatal pathway during PD process. We also speculate the complicated cellular and molecular mechanisms involved in the neuroprotection of parkin in young and old mice model of PD. This work implicates parkin as an important molecule and potential therapeutic candidate in PD.

## Supporting Information

Figure S1 **Levels of TH protein in the striatum of old mice 8 days after MPTP or saline treatment.** Western blot showing striatal TH protein, β-actin served as the loading control (A). Quantification of relative TH protein expression was showed at the bottom (B). Data presented are the means±SE. * p<0.05, significant differences between saline and MPTP-treated mice; n = 3 per group.
(TIF)

Figure S2 **Levels of TH protein in the striatum of wild type and parkin transgenic mice Line P23#.** Samples were collected 1 day after MPTP or saline treatment. Western blot showing striatal TH protein in young (A) and old (B) mice. β-actin served as the loading control. Quantification of relative TH protein expression was showed in the right panel. Data presented are the means±SE. **p<0.01, significant differences between saline and MPTP-treated mice; n  = 4 per group.
(TIF)

Figure S3 **Transcriptional expression of iNOS, TNF-α, CHIP, UCH-L1 and Timm22 in the substantia nigra of mice from experimental groups.** mRNA levels of iNOS (A), TNF-α (B), CHIP (C) and UCH-L1 (D) were determined by real-time PCR and were normalized to GAPDH. Timm22 served as a control (E). Values are means±SE. *p <0.05, significant differences between saline and MPTP-treated mice; #p<0.05, significant differences between wild type and transgenic mice; n = 4–7 per group.
(TIF)

Figure S4 **Levels of Bax, Bcl-2, PINK1, UCH-L1 and DJ-1 protein in the striatum.** Bax, Bcl-2, PINK1, UCH-L1 and DJ-1 protein levels in the striatum after MPTP or saline treatment were detected by western blot.
(TIF)

Table S1 **Summary of the results in the striatum and SN of wild type and Parkin transgenic mice.**
(TIF)
