# Supplementary figures and images for: Overexpression of Parkin Ameliorates Dopaminergic Neurodegeneration Induced by 1- Methyl-4-Phenyl-1,2,3,6-Tetrahydropyridine in Mice
Source: PLoS One. 2012 Jun 29;7(6):e39953. doi: 10.1371/journal.pone.0039953 (PMC3390003; doi:10.1371/journal.pone.0039953)

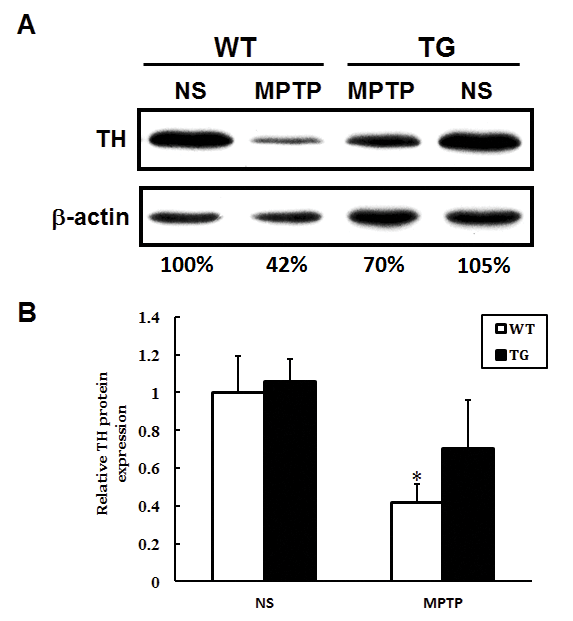

Supplement: Figure S1 — Levels of TH protein in the striatum of old mice 8 days after MPTP or saline treatment. Western blot showing striatal TH protein, β-actin served as the loading control (A). Quantification of relative TH protein expression was showed at the bottom (B). Data presented are the means±SE. * p<0.05, significant differences between saline and MPTP-treated mice; n = 3 per group. (TIF) [file pone.0039953.s001.tif]

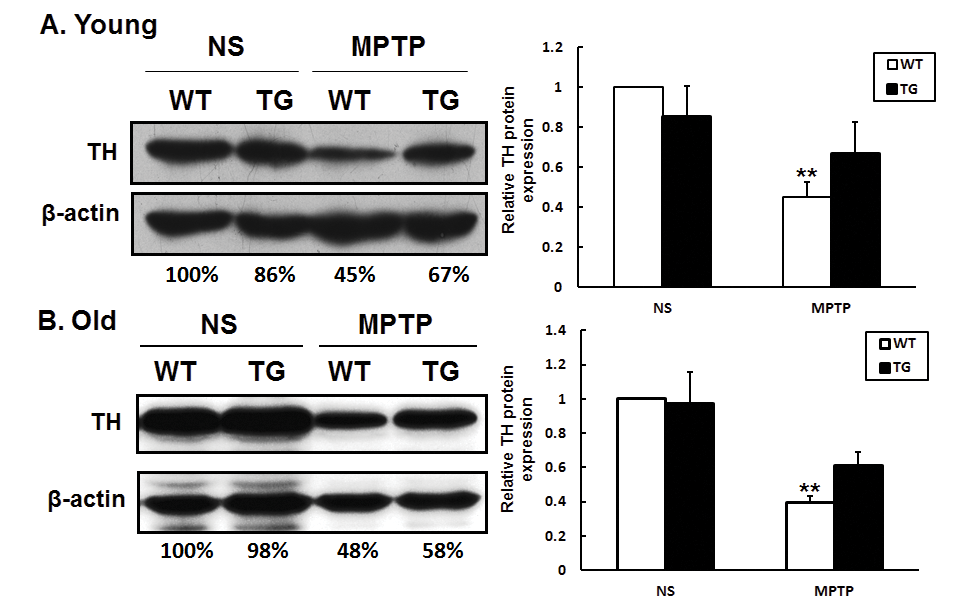

Supplement: Figure S2 — Levels of TH protein in the striatum of wild type and parkin transgenic mice Line P23#. Samples were collected 1 day after MPTP or saline treatment. Western blot showing striatal TH protein in young (A) and old (B) mice. β-actin served as the loading control. Quantification of relative TH protein expression was showed in the right panel. Data presented are the means±SE. **p<0.01, significant differences between saline and MPTP-treated mice; n = 4 per group. (TIF) [file pone.0039953.s002.tif]

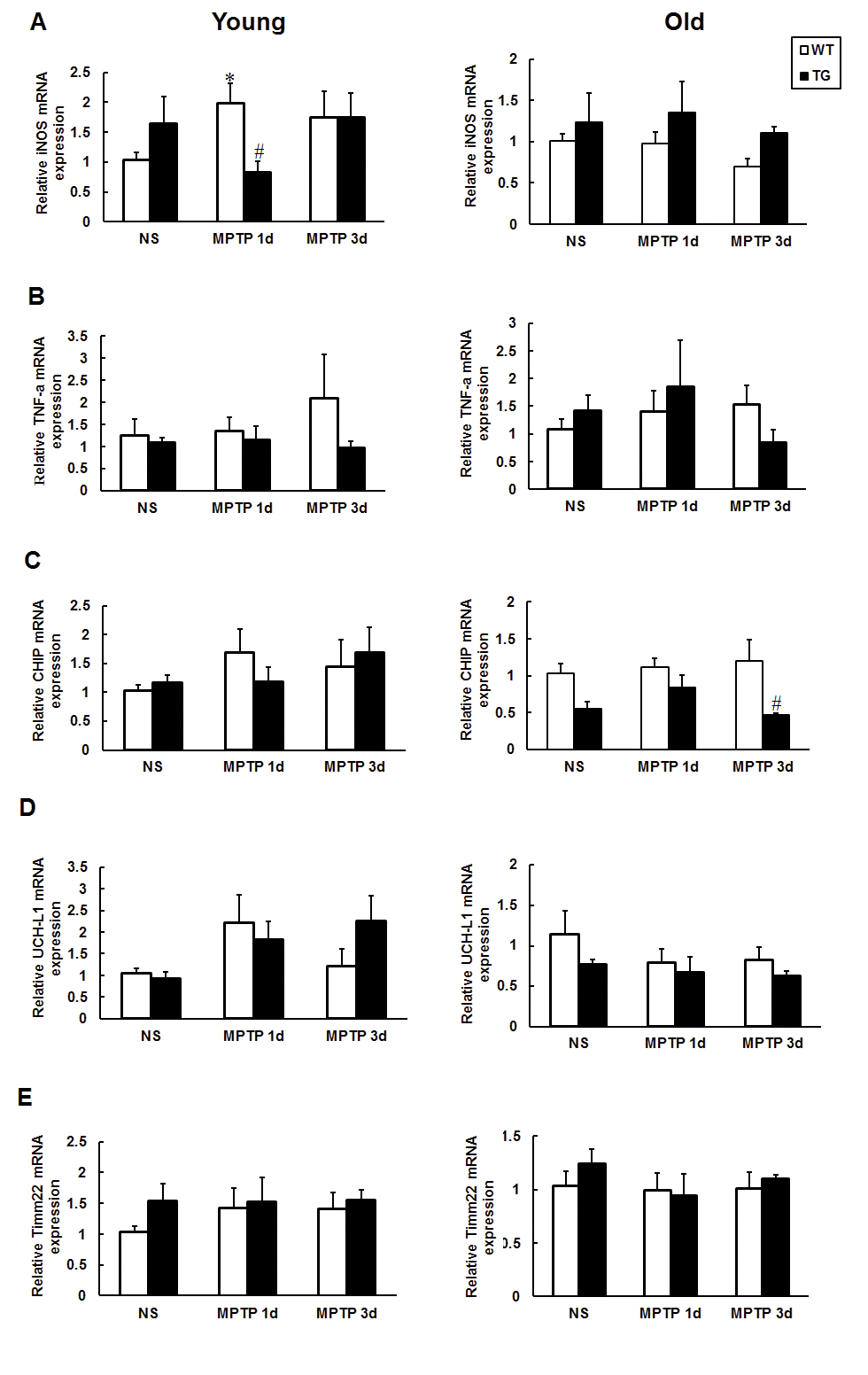

Supplement: Figure S3 — Transcriptional expression of iNOS, TNF-α, CHIP, UCH-L1 and Timm22 in the substantia nigra of mice from experimental groups. mRNA levels of iNOS (A), TNF-α (B), CHIP (C) and UCH-L1 (D) were determined by real-time PCR and were normalized to GAPDH. Timm22 served as a control (E). Values are means±SE. *p <0.05, significant differences between saline and MPTP-treated mice; #p<0.05, significant differences between wild type and transgenic mice; n = 4–7 per group. (TIF) [file pone.0039953.s003.tif]

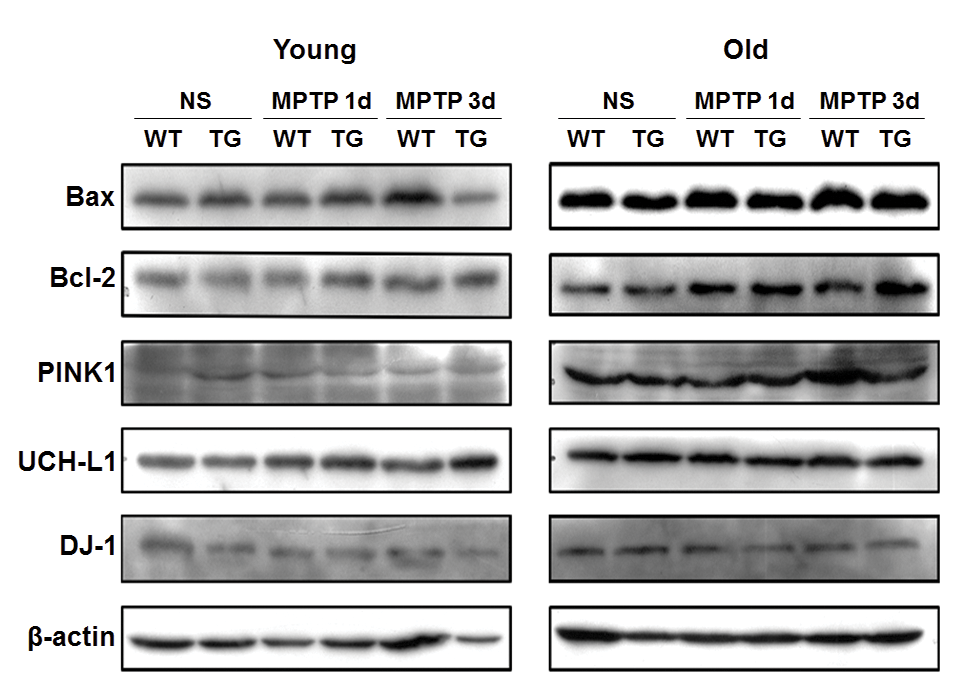

Supplement: Figure S4 — Levels of Bax, Bcl-2, PINK1, UCH-L1 and DJ-1 protein in the striatum. Bax, Bcl-2, PINK1, UCH-L1 and DJ-1 protein levels in the striatum after MPTP or saline treatment were detected by western blot. (TIF) [file pone.0039953.s004.tif]

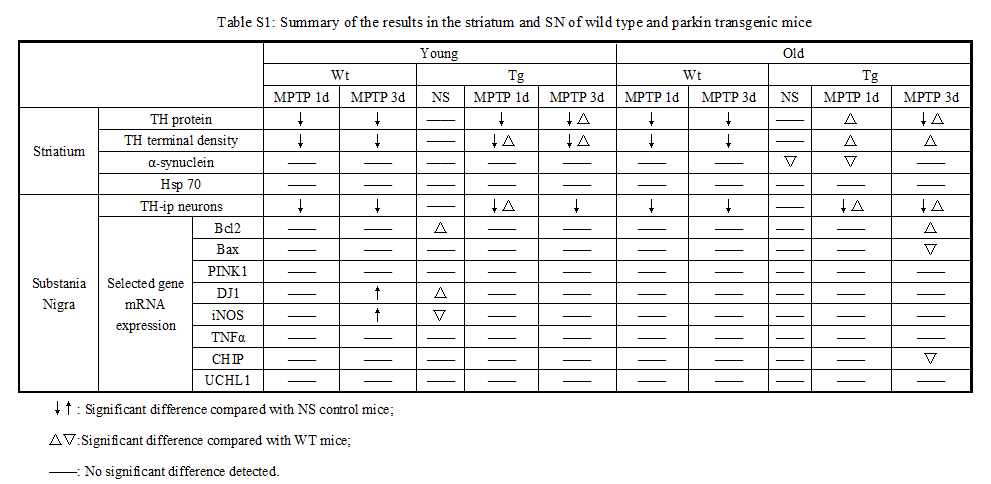

Supplement: Table S1 — Summary of the results in the striatum and SN of wild type and Parkin transgenic mice. (TIF) [file pone.0039953.s005.tif]
